# Failure of available scoring systems to predict ongoing infection in patients with abdominal sepsis after their initial emergency laparotomy

**DOI:** 10.1186/1471-2482-11-38

**Published:** 2011-12-23

**Authors:** Oddeke van Ruler, Jordy JS Kiewiet, Kimberley R Boer, Bas Lamme, Dirk J Gouma, Marja A Boermeester, Johannes B Reitsma

**Affiliations:** 1 Department of Surgery, Academic Medical Center, Amsterdam, The Netherlands; 2 Department of Clinical Epidemiology, Biostatistics and Bioinformatics, Academic Medical Center, Amsterdam, the Netherlands

## Abstract

**Background:**

To examine commonly used scoring systems, designed to predict overall outcome in critically ill patients, for their ability to select patients with an abdominal sepsis that have ongoing infection needing relaparotomy.

**Methods:**

Data from a RCT comparing two surgical strategies was used. The study population consisted of 221 patients at risk for ongoing abdominal infection. The following scoring systems were evaluated with logistic regression analysis for their ability to select patients requiring a relaparotomy: APACHE-II score, SAPS-II, Mannheim Peritonitis Index (MPI), MODS, SOFA score, and the acute part of the APACHE-II score (APS).

**Results:**

The proportion of patients requiring a relaparotomy was 32% (71/221). Only 2 scores had a discriminatory ability in identifying patients with ongoing infection needing relaparotomy above chance: the APS on day 1 (AUC 0.61; 95%CI 0.52-0.69) and the SOFA score on day 2 (AUC 0.60; 95%CI 0.52-0.69). However, to correctly identify 90% of all patients needing a relaparotomy would require such a low cut-off value that around 80% of all patients identified by these scoring systems would have negative findings at relaparotomy.

**Conclusions:**

None of the widely-used scoring systems to predict overall outcome in critically ill patients are of clinical value for the identification of patients with ongoing infection needing relaparotomy. There is a need to develop more specific tools to assist physicians in their daily monitoring and selection of these patients after the initial emergency laparotomy.

**Trial registration number:**

ISRCTN: ISRCTN 51729393

## Background

Our group conducted a trial among patients with abdominal sepsis comparing on-demand versus planned relaparotomy after the initial emergency operation (RELAP trial) [1]. We concluded that the on-demand strategy should be preferred, based on comparable clinical outcomes (12-month mortality 29% vs. 36%; P = 0.22), but a substantial reduction of healthcare utilization and costs [1]. Planned relaparotomy yielded negative findings in 66% of patients and, thus, had no therapeutic effect in these patients. Improvement of patient selection for relaparotomy in the on-demand strategy however is necessary as 31% of these patients also had a negative relaparotomy [1].

The on-demand strategy implies a vigilant observation of the postoperative peritonitis patient. Improvement of outcome may follow improved monitoring following the initial emergency (index) laparotomy and adequate selection of patients with ongoing infection for reintervention. Presently, the on-demand strategy includes reoperation when patients show clinical deterioration or do not improve [1]. However, these conditions are not well defined. There is no consensus or guideline on patient monitoring to assist treating physicians in the selection of patients for reoperation.

Several commonly used scoring systems exist assessing the severity of disease in critically ill patients by predicting mortality. As these prognostic scores are widely incorporated in the daily treatment of ICU patients, we questioned whether they would be useful in selecting patients with ongoing abdominal infection needing a relaparotomy or reintervention (by relaparotomy or by percutaneous drainage).

## Methods

### Study population

The RELAP trial was a randomized controlled trial comparing relaparotomy on-demand with planned relaparotomy in patients with severe abdominal sepsis (APACHE-II > 10) receiving an emergency laparotomy. Details on inclusion criteria were reported in the article presenting the main results [1]. The 'on-demand' strategy was defined as performing a relaparotomy only in case of clinical deterioration or lack of improvement, monitored by physiological, laboratory and radiological parameters. The planned strategy was defined as performing a relaparotomy every 36 to 48 hours until the abdomen was macroscopically clean at the beginning of the final relaparotomy [1].

The trial cohort consisted of 229 patients of which 114 were randomly allocated to the on-demand strategy and 115 to the planned strategy. For the present study we included patients from both arms, but excluded those patients in whom the first relaparotomy took place more than 7 days after the initial emergency operation (n = 8). We maintained this 7-day period as ongoing infection directly associated with the primary disease, is presumed to occur within this time window. Moreover, the essential difference between the planned and on-demand strategy lies in the first relaparotomy being performed within two or three days after index surgery in the planned strategy. Commitment for relaparotomy on the second or third day is not part of the on-demand strategy, but reconciliation of the need for relaparotomy is made day by day in particular this first week.

#### Outcomes

##### Ongoing infection needing relaparotomy

In the planned strategy group 'ongoing infection needing a relaparotomy' was defined as positive macroscopic findings at relaparotomy. Moreover, all planned patients who died within 14 days were classified as 'ongoing infection'. Death within this period was all cause. Within this short time frame from emergency laparotomy mortality was classified as due to (deterioration from) underlying abdominal sepsis. A negative relaparotomy (no residual infection or new pathology) was classified as 'no ongoing infection, not needing relaparotomy'.

**Figure 1:**
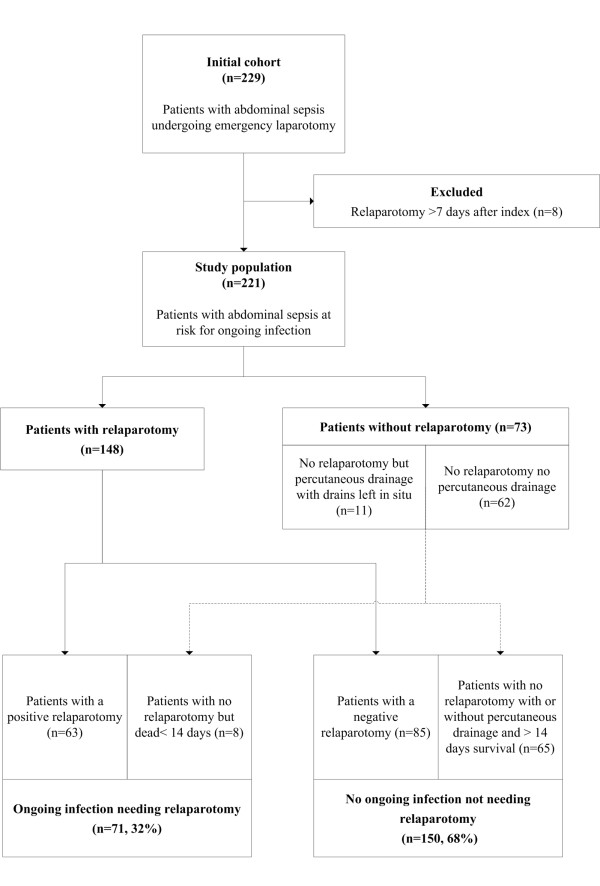
**Flow chart showing patient selection and outcome definition**.

In the on-demand treated patients only 48% received a first relaparotomy. These patients were classified as 'ongoing infection, needing a relaparotomy' in case of positive macroscopic findings at relaparotomy. In the 52% of on-demand patients not receiving relaparotomy obviously no direct visual abdominal inspection was performed. Therefore, we used a 14-day follow-up period as additional verification: a patient without visual verification that died within this period was classified as 'ongoing infection needing relaparotomy'. Patients without visual verification who survived at least 2 weeks after index surgery were classified as 'no ongoing infection, not needing relaparotomy' - assuming that if these patients would have received an early relaparotomy, findings would have been negative (Figure 1).

### Ongoing infection needing reintervention

Patients needing reintervention (n = 82) included all patients that were classified as 'ongoing infection needing relaparotomy' supplemented by patients that received US- or CT-guided percutaneous drainage (PCD) of abdominal fluid with placement of drains for continuous drainage and lavage (n = 11; Figure 1).

### Scoring systems

Widely-used scoring systems were evaluated for predicting the need of relaparotomy after index operation. Scoring systems evaluated were the Acute Physiology and Chronic Health Evaluation (APACHE)-II score [2], the simplified Acute Physiology Score (SAPS)-II [3], the Mannheim Peritonitis Index (MPI) [4,5], the Multiple Organ Dysfunction Score (MODS) [6], the Sepsis-related Organ Failure Assessment (SOFA) score [7], and the APS, the physiological part extracted from the APACHE-II score [2].

The APACHE-II score was assessed, following the RELAP trial protocol, using the worst values of each independent constituent in a 24-hour time frame including the index laparotomy [1]. The SAPS-II and MPI were compiled by adding worst values of independent constituents in the initial 24 hours following index laparotomy.

Sequential scores, SOFA, APS and MODS were calculated on day 1 and 2, following index laparotomy by adding worst values of independent constituents measured that day (Table 1).

**Table 1:** Overview of components of the various existing scoring systems.

	**APACHE-II**	**SAPS-II**	**MPI**	**SOFA**	**MODS**	**APS**
	
Type of admission		*√*				
Age	*√*	*√*	*√*			
Gender			*√*			
Chronic disease present	*√*	*√*				
Malignant comorbidity			*√*			
Organ failure present			*√*			
Temperature	*√*	*√*				*√*
White blood cell counts		*√*				*√*
Oxygenation^a^	*√*	*√*		*√*	*√*	*√*
Mechanical ventilation		*√*				
Respiratory rate	*√*					*√*
Arterial pH	*√*					*√*
HCO3^-^		*√*				
Cardiovascular state^b^				*√*		
Heart rate	*√*	*√*			*√*	*√*
Blood pressure^c^	*√*	*√*		*√*	*√*	*√*
Hematocrit	*√*					*√*
Creatinine	*√*			*√*	*√*	*√*
Urine output		*√*		*√*		
Urea		*√*				
Bilirubin		*√*		*√*	*√*	
Coagulation (thrombocytes)				*√*	*√*	
Potassium	*√*	*√*				*√*
Sodium	*√*	*√*				*√*
Glasgow coma score	*√*	*√*		*√*	*√*	
Disease specific parameters^d^			*√*			

^a ^PaO_2 _or PaO_2_/FiO_2 _ratio

^b ^includes administration and dosage of (nor)epinephrine, dopamine or dobutamine

^c ^systolic blood pressure, mean arterial pressure (MAP) or as part of the pressure adjusted heart rate (PAR)

^d ^disease specific parameters include duration of peritonitis, origin, extent of peritonitis and type of contamination.

#### Statistical analysis

##### Baseline characteristics

Demographic data, clinical characteristics and findings at index operation were compared between patients who did or did not have ongoing infection needing relaparotomy. Continuous variables were expressed as mean ± standard deviation or median (25-75% interquartile range) and compared, respectively, using Student's t-test or Mann-Whitney U-test depending on the skewness of the data. Categorical variables were reported as absolute numbers (frequency with percentages) and analyzed using the χ^2 ^test.

##### Predicting 'ongoing infection needing relaparotomy'

To asses the ability of existing scoring systems to identify patients with ongoing infection needing relaparotomy we focused on the area under the receiver operating characteristic (ROC) curve (AUC). AUC's are presented for the APACHE-II, SAPS-II and MPI at index laparotomy. AUC's are presented for the MODS, SOFA score, and APS at day 1, day 2, and the absolute difference between day 2 and day 1. Patients who were already dead and patients who already received a relaparotomy prior to the day of the measurements were not included in the analyses.

Logistic regression models were used to calculate these AUC's, also known as the concordance of c-statistics. These logistic regression models also provide odds ratios and 95% confidence intervals (CI) expressing the strength of association between a risk score and the probability of ongoing infection needing relaparotomy.

Although we tried to harmonize the definition of ongoing infection needing relaparotomy across the two surgical strategies, we specifically examined whether there was a difference in predictive capability of one of the existing scoring system between patients treated by either surgical strategy. To examine this, logistic regression models were constructed containing type of surgical strategy, scoring system, and interaction between surgical strategy and scoring system. A significant P-value for the interaction term would indicate that the predictive ability of such a scoring system was different between surgical strategies, and probably related to a difference in defining the outcome.

If a scoring system had a significantly better discriminatory ability than can be expected by chance only (AUC > 0.6), we calculated a specific cut-off point that would have led to a sensitivity of 90%. Applying that cut-off value would have identified 90% of all patients needing relaparotomy and consequently miss 10% of these patients. Such a cut-off analysis shows the consequences of applying a specific cut-off value and in particular it reveals the number of patients not needing a relaparotomy who would have been operated based upon a score above the cut-off value (1-specificity).

### Predicting 'ongoing infection needing reintervention'

To assess the ability of existing scoring systems to identify patients with ongoing infection using the wider definition of 'needing reintervention', we determined the AUC's as measure of predictive value for 'ongoing infection needing reintervention' [8].

### Predicting mortality

To verify consistency of data we examined the ability of examined scoring systems to predict mortality, the very purpose for which they were originally developed and validated. We again used AUC's to express the ability of each score to discriminate between patients who died in-hospital and those who survived their hospital admission [8].

### Missing values

The various existing scoring systems were based on a multitude of different variables that had to be recorded prospectively per protocol on consecutive days upon inclusion of the RELAP trial. There were inevitably missing values in our dataset. Data checks were performed to detect any available missing values.

Although we had a low rate of missing values, in a compository score it would introduce uncertainty even when values of other variables were present. Therefore, we used multiple imputation to replace missing values with a set of plausible values that represent the uncertainty around the right value to impute. The multivariate relationships between all underlying variables were used to impute missing values (Markov Chain Monte Carlo, SAS). Appropriate transformations were applied to individual variables to improve normality [9,10]. For patients not admitted to the ICU at day 1 and/or day 2 normal values were imputed for components specifically associated with ICU care (central venous pressure, air oxygen pressure (FiO_2_) in case of no oxygen suppletion, arterial oxygenation (PaO_2_), and Glascow coma scale) [11].

The multiple imputed data sets were analyzed (one by one) using standard procedures (e.g. logistic regression) for complete data. Then the results from these analyses were combined to produce estimates and confidence intervals that properly reflect the uncertainty due to missing values. We used a total of ten rounds of imputation to estimate the final parameters with their confidence intervals [9,10,12-14].

P-values less than 0.05 were considered statistically significant. Statistical analyses were carried out using SAS 9.1 (SAS Institute, Cary, NC, USA).

## Results

### Patient inclusion

The study population consisted of 221 patients at risk for ongoing infection after emergency laparotomy because of secondary peritonitis. Figure 1 shows that using the specified outcome definition 71 patients (32%) were classified as 'ongoing infection needing relaparotomy'.

For the sequential scoring systems, 3 patients were excluded from the analyses at day 1, because 1 patient had a relaparotomy within several hours of the index laparotomy and 2 patients had died. For analyses at day 2 some more patients were excluded from analyses: 12 patients had already undergone a relaparotomy and 1 patient had died, leaving 205 patients available for analyses (needing relaparotomy n = 64).

Table 2 lists the demographic and baseline characteristics of both outcome groups. More than 90% of patients had been admitted to the ICU. As can be expected, patients classified as 'ongoing infection needing relaparotomy' had a significant longer ICU stay and high mortality rate.

**Table 2:** Demographic, initial peritonitis and recovery data for all 221 included patients.

**Characteristic**	**Ongoing infection needing relaparotomy (n = 71)**		**No ongoing infection, not needing relaparotomy (n = 150)**		**P-value**
	
Median age (IQR)	69	(57 to 74)	70	(57 to 76)	0.36
					
Male n (%)	38	(54%)	65	(43%)	0.16
					
Postoperative peritonitis n (%)	32	(45%)	71	(47%)	0.75
					
Generalized peritonitis n (%)	47	(66%)	88	(59%)	0.48
					
ICU admission n (%)	69	(97%)	135	(90%)	0.061
					
Length of ICU stay	12	(7 to 32)	7	(4 to 16)	0.001
					
Length of index hospital stay	32	(17 to 74)	28	(17 to 49)	0.28
					
In-hospital mortality n (%)	26	(37%)	23	(15%)	< 0.001

### Predicting 'ongoing infection needing relaparotomy'

**Figure 2:**
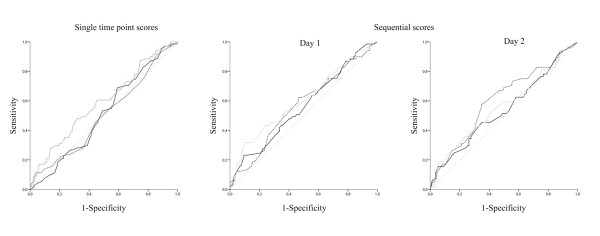
**Receiver operating characteristic (ROC) curves associated with the area under the curves depicted in Table 3**. ROC's are displayed for the scoring systems measured at a single time point (APACHE-II(), SAPS-II(), MPI() compared to the reference line ()) and for sequential scoring systems (MODS(), SOFA(), APS() compared to the reference line ()) measured at day 1 and at day 2.

None of the interactions between existing scoring systems and the applied surgical strategy (on-demand or planned relaparotomy strategy) were significant with P-values ranging from 0.098 to 0.982. Therefore, all ROC analyses were based on the total cohort of patients from the RELAP trial (Table 3). The ROC curves showing pairs of sensitivity and specificity for possible cut-off values are presented in Figure 2.

**Table 3:** Mean scores with associated standard deviations (SD) compared for patients with 'ongoing infection needing relaparotomy' (n = 71) and patients with 'no ongoing infection, not needing relaparotomy' (n = 150).

**Score**	**Ongoing infection [Mean score (SD)]**	**No ongoing infection, [Mean score (SD)]**	**AUC (95% CI)**	**P-value**^**a **^
	
APACHE-II	16.1 (4.8)	15.8 (4.0)	0.50 (0.42 to 0.58)	0.961
SAPS-II	40.2 (12.3)	36.0 (11.3)	0.59 (0.51 to 0.67)	0.033
MPI	26.8 (6.9)	26.5 (7.9)	0.51 (0.43 to 0.59)	0.824
SOFA (day 1)^b^	7.3 (3.8)	6.4 (3.4)	0.57 (0.49 to 0.65)	0.108
SOFA (day 2)^c^	6.8 (3.8)	5.8 (3.4)	0.60 (0.52 to 0.69)	0.017
SOFA Delta (day 2-1)^c^	-0.17 (2.2)	-0.93 (2.3)	0.58 (0.50 to 0.66)	0.073
MODS (day 1)^b^	5.0 (3.0)	4.3 (2.7)	0.56 (0.48 to 0.64)	0.141
MODS (day 2)^c^	4.2 (3.0)	3.7 (2.6)	0.54 (0.45 to 0.63)	0.349
MODS Delta (day 2-1)^c^	-0.48 (1.8)	-0.60 (1.8)	0.52 (0.43 to 0.60)	0.726
APS (day 1)^b^	8.3 (4.6)	6.6 (3.6)	0.61 (0.52 to 0.69)	0.012
APS (day 2)^c^	6.8 (3.7)	5.9 (3.2)	0.56 (0.47 to 0.65)	0.154
APS Delta (day 2-1)^c^	-1.03 (3.7)	-0.64 (3.7)	0.55 (0.46 to 0.63)	0.277

^a ^P-value wether different from an AUC of 0.50

^b ^patients deceased on day 0 (n = 2) and patient receiving relaparotomy on day 0 (n = 1) excluded

^c ^patients deceased on day 0 and 1 (n = 3) and patients receiving relaparotomy on day 0 and 1 (n = 13) excluded

The single time point severity of disease scores, APACHE-II score, SAPS-II and MPI showed no predictive value for 'ongoing infection needing relaparotomy' with AUC's all below 0.6 (Table 3, Figure 2).

The SOFA score had no predictive value on day 1 (AUC 0.57, 95%CI 0.49-0.65) but improved to modest on day 2 (AUC 0.60, 95%CI 0.52-0.69; Table 3, Figure 2). The absolute difference (delta SOFA measurements between day 2 and day 1) showed no discriminatory value. The MODS also showed no discriminatory value for ongoing infection on either consecutive day (Table 3, Figure 2).

The APS, however, showed a modest discriminatory ability at day 1 with an AUC of 0.61 (95%CI 0.52-0.69), at day 2 the AUC was 0.56 (95%CI 0.47-0.65; Table 3, Figure 2).

### 90% sensitivity

For scoring systems that performed significantly better than chance, we determined a cut-off score that would produce a sensitivity of 90% and then calculated the proportion of patients undergoing an unnecessary relaparotomy (1-specificity).

A score of 3.1 determined for the APS would have identified 90% of the patients requiring a relaparotomy, but the corresponding specificity was only 17%. Based on this cut-off value, the positive predictive value would have been 33%, indicating that for all 186 patients with a score above this cut-off of 3.1 only 62 patients would have been rightfully reoperated, and 124 patients would have been reoperated under suspicion of ongoing abdominal infection but with negative findings at relaparotomy. The negative predictive value, based on this cut-off value, would have been 78%, indicating that 25 of the 32 patients with a score under the cut-off value of 3.1 would rightfully not have been reoperated. However, 7 patients with ongoing infection needing relaparotomy would have been withheld from relaparotomy (Table 4).

**Table 4:** Performance of scoring systems, moderately predictive for 'ongoing infection needing relaparotomy' (AUC > 6.0; SOFA day 2 and APS day 1), in selecting patients for relaparotomy using a cut-off value based on a 90% sensitivity.

		Relaparotomy required		
		**Yes**	**No**	
			
**SOFA**	Above 1.4	57	122	PPV^a ^= 32%
**cut-off**	Below 1.4	7	19	NPV^b ^= 73%
**(range score 0-18)**		Sensitivity = 90%	Specificity = 13%	
	*total*	*64*	*141*	
				
		**Yes**	**No**	
			
*APS*	Above 3.1	62	124	PPV^a ^= 33%
**cut-off**	Below 3.1	7	25	NPV^b ^= 78%
**(range score 0-56)**		Sensitivity = 89%	Specificity = 17%	
	*total*	*69*	*149*	
				

^a ^positive predictive value

^b ^negative predictive value

The 90% sensitivity cut-off for day 2 SOFA scores (1.4) would have a positive predictive value of 32% and a negative predictive value of 73% (Table 4).

### Predicting ongoing infection needing reintervention

Eighty-two patients were classified as 'ongoing infection needing reintervention' (Figure 1). Only the APS at day 1 showed an AUC 0.6 for prediction of 'ongoing infection needing reintervention' (95%CI 0.52-0.68; Table 5).

**Table 5:** Predictive value comparing patients needing a reintervention (n = 82) with not needing reintervention and for inhospital mortality (n = 49).

	**Score**	**AUC (95% CI)**	**P-value whether AUC different from 0.5**
	
**Reintervention (n = 82)**			
	APACHE-II	0.49 (0.41 to 0.57)	0.860
	SAPS-II	0.56 (0.48 to 0.64)	0.145
	MPI	0.52 (0.44 to 0.60)	0.666
	SOFA (day 1) ^a^	0.55 (0.47 to 0.63)	0.205
	SOFA (day 2) ^b^	0.57 (0.49 to 0.65)	0.088
	MODS (day 1) ^a^	0.55 (0.47 to 0.63)	0.199
	MODS (day 2) ^b^	0.53 (0.45 to 0.61)	0.469
	APS (day 1)a	0.60 (0.52 to 0.68)	0.019
	APS (day 2) ^b^	0.53 (0.44 to 0.61)	0.531
			
**Inhospital mortality (n = 49)**			
	APACHE-II	0.74 (0.65 to 0.82)	< 0.001
	SAPS-II	0.80 (0.73 to 0.87)	< 0.001
	MPI score	0.60 (0.52 to 0.69)	0.026
	SOFA (day 1) ^c^	0.72 (0.63 to 0.81)	< 0.001
	MODS (day 1) ^c^	0.76 (0.68 to 0.83)	< 0.001
	APS (day 1) ^c^	0.68 (0.59 to 0.77)	< 0.001

^a ^patients deceased on day 0 (n = 2) and patient receiving relaparotomy on day 0 (n = 1) excluded

^b ^patients deceased on day 0 and 1 (n = 3) and patients receiving relaparotomy on day 0 and 1 (n = 13) excluded

^c ^patients deceased on day 0 (n = 2) and patient receiving relaparotomy on day 0 (n = 1) excluded

### Predicting mortality

All evaluated scoring systems were predictive of mortality in peritonitis patients, as score AUC's were significantly different from an AUC of 0.5 (Table 5).

## Discussion

The predictive value of available scoring systems, in particular those that can be assessed sequentially, for ongoing abdominal infection needing relaparotomy is not known. In clinical practice changes in organ functions are seen as useful triggers to expand diagnostic tools or intervention. However, only the SOFA score and APS had equally modest discriminatory ability for predicting ongoing infection needing relaparotomy. Furthermore, they showed an extremely low specificity for a 90% sensitivity. Broadening the definition of ongoing abdominal infection by patients needing reintervention (relaparotomy or percutaneous drainage) did not enhance identification of patients with persistent peritonitis.

The RELAP trial concludes that the on-demand strategy is preferred [1]. Stringent monitoring of patients is a vital component of this strategy. A scoring system can aid in adequate and timely identification of patients for relaparotomy. Ideally, such a prediction model should be a sequential score. Changes in organ failure may be of better value in objectifying the clinical course of the disease, in particular since postoperative (follow-up) variables are more predictive than variables that become available during index laparotomy [15].

We were surprised by the low performance of these well-known scoring systems, as most of these scores quantify organ function. However, none of the evaluated scoring systems were originally developed to predict the need of a relaparotomy for ongoing peritonitis following emergency laparotomy in the acute phase of the disease [2-7]. All scores, except the MPI, have been developed to predict death for ICU patients in general and for groups of patients (strata) rather than predicting death for individual patients [2,3,6,7]. Although the MPI is specifically developed for patients with abdominal sepsis, it is focused on prediction of death rather than occurrence of ongoing infection [16]. Also, the MPI largely consists of peritonitis-related data, determined at the initial emergency laparotomy [5]. These variables are described to be less predictive than physiological post-operative variables [15]. All scores, indeed, did better at predicting death, as they are developed and validated to do.

Prognostic relevance of the SOFA score in combination with inflammatory parameters was also found in a recent study conducted by Zügel *et al*., even though results were based on only a small number of events [17]. Torer *et al. *and Tan *et al. *identified possible prognostic relevance for the MPI in retrospective cohorts with patients with secondary peritonitis due to postoperative complications and community acquired perforations of small bowel and colon [17-20]. However, quantification of or changes in organ failure does not seem to differentiate between ongoing organ failure due to abdominal sepsis despite source control of the initial causative focus and ongoing abdominal infection.

For the non-reoperated patients, the time frame in which the predictor status of the sequential scoring systems was assessed was less precise. Our best deduction was to evaluate the scores during the clinical phase in which the dilemma of early relaparotomy is most prominent; day 1 and day 2 after the initial emergency laparotomy, for all included patients. In view of the disappointing predictive values, it is unlikely that extension of assessment after day 2 would have revealed completely different results.

Patients included in this study were randomized to the on-demand or the planned strategy [1]. This enhances the generalizability of the results but foremost eliminates selection bias in choice of practiced treatment strategies. Differences in these treatment arms did lead to differential verification, but not necessarily to verification bias. Another option would have been to use only the planned arm of the trial, as all these patients were reoperated and had uniform outcome verification. Instead, all existing scoring systems were tested for the assumption that both the on-demand and planned strategy could be combined for the above analyses and we found no significant interaction between treatment strategies and predictive ability of the various scoring systems. This means that the predictive ability of existing scoring systems is comparable for both on-demand and planned treated patients. Importantly, also the proportion of events (positive findings at relaparotomy) was comparable for both strategies (29% for on-demand vs. 32% for planned) [1].

For clinical purposes, discriminatory power is more important than stratification. A 90% level of sensitivity was employed, as it is considered worse to mistakenly not re-operate a patient with ongoing infection needing relaparotomy than it is to reoperate a patient on the suspicion of ongoing infection but with negative findings [15,21]. The approximate of 90% sensitivity was chosen to determine a cut-off for adequate scoring systems, reflecting the trade-off between a false positive prediction of peritonitis (negative relaparotomy) and a false negative prediction of peritonitis (no relaparotomy although one is needed). Nevertheless, performing too many negative relaparotomies should be avoided [22]. None of the scoring systems had a clinical important predictive value nor demonstrated a clinically useful discriminatory ability. In order not to withhold relaparotomy from too many patients who need treatment for ongoing infection, an unacceptable high proportion of inappropriate relaparotomies would be performed based on the scores.

All presented, existing scoring systems lack the additional information derived from diagnostic imaging techniques which is likely valuable for selection of patients with ongoing infection needing reintervention. For patients suspected of abdominal infection following elective abdominal surgery, CT imaging has a high diagnostic accuracy [23]. The exact value of diagnostic imaging in operated peritonitis patients with suspected ongoing abdominal infection is not known, as consequences from management decisions based on CT results have not been evaluated yet. Future research is needed to determine the exact accuracy of CT scanning in on-demand treated peritonitis patients who are suspected of ongoing infection.

## Conclusions

None of the existing and widely used severity-of-disease scores, specifically developed for critically ill patients, was clinically useful in the identification of patients with ongoing infection needing a relaparotomy. Therefore, new tools need to be developed that specifically incorporate parameters indicative for ongoing abdominal infection, rather than merely ongoing organ failure, in patients with abdominal sepsis. Preferentially, these specific tools combine clinical findings, laboratory measurements and results from diagnostic imaging tests to assist the multidisciplinary team in selecting patients for reintervention to treat ongoing abdominal infection.

## Competing interests

The authors declare that they have no competing interests.

## Authors' contributions

All authors have read and approved the final manuscript. OR designed the study, collected, analyzed and interpreted data. Furthermore she wrote the manuscript. JK analyzed and interpreted data, and drafted the manuscript. KB analyzed and interpreted data, and drafted the manuscript. BL helped to collect data and drafted the manuscript. DG designed the study and drafted the manuscript. MB designed the study, interpreted data, and drafted the manuscript. JR analyzed and interpreted data, and drafted the manuscript.

## Pre-publication history

The pre-publication history for this paper can be accessed here:

http://www.biomedcentral.com/1471-2482/11/38/prepub
